# A non-invasive 25-Gene PLNM-Score urine test for detection of prostate cancer pelvic lymph node metastasis

**DOI:** 10.1038/s41391-023-00758-z

**Published:** 2024-02-02

**Authors:** Jinan Guo, Liangyou Gu, Heather Johnson, Di Gu, Zhenquan Lu, Binfeng Luo, Qian Yuan, Xuhui Zhang, Taolin Xia, Qingsong Zeng, Alan H. B. Wu, Allan Johnson, Nishtman Dizeyi, Per-Anders Abrahamsson, Heqiu Zhang, Lingwu Chen, Kefeng Xiao, Chang Zou, Jenny L. Persson

**Affiliations:** 1 Department of Urology, Shenzhen People’s Hospital (The Second Clinical Medical College, Jinan University), Shenzhen, China; 2 Shenzhen Clinical Research Centre for Geriatrics, Shenzhen People’s Hospital, Shenzhen, China; 3 Shenzhen Urology Minimally Invasive Engineering Center, Shenzhen, China; 4 Shenzhen Public Service Platform on Tumor Precision Medicine and Molecular Diagnosis, Clinical Medicine Research Centre, Shenzhen, China; 5 The First Affiliated Hospital, Southern University of Science and Technology, Shenzhen, China; 6 Department of Urology, The Third Medical Centre, Chinese PLA General Hospital, Beijing, China; 7 Olympia Diagnostics, Inc., Sunnyvale, CA USA; 8 Department of Urology, The First affiliated Hospital of Guangzhou Medical University, Guangzhou, China; 9 The University of Hong Kong-Shenzhen Hospital, Shenzhen, China; 10 Department of Bio-diagnosis, Institute of Basic Medical Sciences, Beijing, China; 11 Department of Urology, Foshan First People’s Hospital, Foshan, China; 12 Department of Urology, The First Affiliated Hospital of Sun Yat-Sen University, Guangzhou, China; 13 Clinical Laboratories, San Francisco General Hospital, San Francisco, CA USA; 14 Kinetic Reality, Santa Clara, USA; 15 Department of Translational Medicine, Lund University, Clinical Research Centre, Malmö, Sweden; 16 Key Laboratory of Medical Electrophysiology of Education Ministry, School of Pharmacy, Southwest Medical University, Luzhou, China; 17 Department of Molecular Biology, Umeå University, Umeå, Sweden; 18 Department of Biomedical Sciences, Malmö University, Malmö, Sweden

**Keywords:** Diagnostic markers, Prostate cancer, Cancer therapy

## Abstract

**Background:**

Prostate cancer patients with pelvic lymph node metastasis (PLNM) have poor prognosis. Based on EAU guidelines, patients with >5% risk of PLNM by nomograms often receive pelvic lymph node dissection (PLND) during prostatectomy. However, nomograms have limited accuracy, so large numbers of false positive patients receive unnecessary surgery with potentially serious side effects. It is important to accurately identify PLNM, yet current tests, including imaging tools are inaccurate. Therefore, we intended to develop a gene expression-based algorithm for detecting PLNM.

**Methods:**

An advanced random forest machine learning algorithm screening was conducted to develop a classifier for identifying PLNM using urine samples collected from a multi-center retrospective cohort (*n* = 413) as training set and validated in an independent multi-center prospective cohort (*n* = 243). Univariate and multivariate discriminant analyses were performed to measure the ability of the algorithm classifier to detect PLNM and compare it with the Memorial Sloan Kettering Cancer Center (MSKCC) nomogram score.

**Results:**

An algorithm named 25 G PLNM-Score was developed and found to accurately distinguish PLNM and non-PLNM with AUC of 0.93 (95% CI: 0.85–1.01) and 0.93 (95% CI: 0.87–0.99) in the retrospective and prospective urine cohorts respectively. Kaplan–Meier plots showed large and significant difference in biochemical recurrence-free survival and distant metastasis-free survival in the patients stratified by the 25 G PLNM-Score (log rank *P* < 0.001 and *P* < 0.0001, respectively). It spared 96% and 80% of unnecessary PLND with only 0.51% and 1% of PLNM missing in the retrospective and prospective cohorts respectively. In contrast, the MSKCC score only spared 15% of PLND with 0% of PLNM missing.

**Conclusions:**

The novel 25 G PLNM-Score is the first highly accurate and non-invasive machine learning algorithm-based urine test to identify PLNM before PLND, with potential clinical benefits of avoiding unnecessary PLND and improving treatment decision-making.

## Introduction

Pelvic lymph node metastasis (PLNM) occurs in approximately 15% of prostate cancer (PCa) patients at diagnosis [1]. Although PLNM itself may not cause mortality, patients with PLNM have poor prognosis with shorter recurrence-free survival and cancer-specific survival [2–4]. A study of PCa patients undergoing radical prostatectomy and pelvic lymph node dissection (PLND) showed shorter 10-year cancer-specific survival in patients with PLNM than patients without PLNM [5]. Other studies found that patients with PLNM had higher incidence of recurrence after radical prostatectomy and radiation therapy [2–4]. PLNM is an important prognostic factor for biochemical recurrence (BCR), distant metastasis, and patient survival, therefore, it plays an important role in PCa treatment. Accurate identification of PLNM before treatment can assist clinical treatment decision-making and prediction of treatment outcome.

According to the NCCN guidelines (https://www.nccn.org/guidelines/guidelines-detail?category=1&id=1459), patients with high cancer risk (intermediate-, high- and very-high-risk) are recommended to receive PLND or extended PLND (ePLND) during radical prostatectomy (RP) if the predicted probability of PLNM by nomograms is ≥2%, whereas low cancer risk (very low-, and low-risk) patients receive no PLND/ePLND. Based on EAU guidelines (https://uroweb.org/guidelines/prostate-cancer), patients with ≥5% risk are recommended to have ePLND. Although the therapeutic benefit of ePLND is controversial, with studies showing little improvement in the risk of BCR, cancer-specific mortality, or distant metastasis [6], this result could be due to the fact that most patients undergoing ePLND in the study had low risk cancer or no PLNM. This is supported by other studies showing survival benefit of ePLND in patients with pN1 and high cancer risk [4, 7–10]. Nevertheless, PLND is considered as the best method for determining PCa N stage for treatment decision-making [11, 12]. However, the benefit of PLND is offset by serious perioperative complications, such as infection, seroma near the incision site, pain or numbness due to nerve damages, and lymphedema [13]. Thus, to avoid unnecessary PLND/ePLND and the potential side effects, it is essential to accurately identify patients with PLNM before the treatment.

However, current methods to identify/predict PLNM in clinical practice, including nomograms, clinicopathological parameters, imaging tools, and predictive models using artificial intelligence/machine learning algorithms to analyze imaging results, have limited accuracy with low sensitivity and low to moderate area under the ROC curve (AUC) [14–20]. The nomograms to predict PLNM risk, such as the Briganti score and the Memorial Sloan Kettering Cancer Center (MSKCC) score, have been shown to have moderate predictive accuracy with AUC below 0.80 [14–16]. Multiparametric magnetic resonance imaging (mpMRI) is widely used to detect PLNM for tumor and nodal stage with low sensitivity of 40–60% [17, 21]. To improve imaging interpretation of nodal stage and diagnostic accuracy, imaging data analysis using machine learning or deep learning approaches have been developed, resulting in higher accuracy than nomograms [18, 19]. PSMA-PET/CT coupled with data analysis by convolutional neural networks has also been used to identify PLNM with improved AUC, yet is still limited by the size of PLNM that can be detected [21]. In addition, multimodal predictive signatures combining imaging measurements with clinicopathological factors are being developed to improve PLNM detection [20, 22]. A nomogram incorporating MRI-targeted biopsy and clinicopathological factors using a risk cutoff threshold of 7% was found to identify extended lymph node dissection (eLND) with AUC of 0.79 [23]. However, none of these methods possess high accuracy with sensitivity and specificity over 90% and AUC above 0.9, resulting in a large number of non-PLNM patients undergoing unnecessary PLND and many PLNM patients missing PLND.

Thus, there is an unmet medical need to develop more accurate tests for selecting PLNM patients for PLND/eLND. Many genes are involved in PCa progression and metastasis, so using multiple genes important for these processes may provide a more accurate detection method.

In this study, we intended to develop a non-invasive, urine-based gene classifier for detecting/predicting PLNM. Its diagnostic performance was assessed and validated in two independent multicenter urine study cohorts.

## Methods

### Retrospective and prospective urine studies

A multi-center retrospective urine study was approved by the Institutional Review Board (IRB) of San Francisco General Hospital (San Francisco, USA) (IRB #: 15-15816) and conducted at San Francisco General Hospital to use archived urine sediments to develop and validate urine biomarkers for detecting prostate cancer (PCa) lymph node metastasis (PLNM). The prospectively designed and retrospectively collected pre-biopsy urine samples were selected randomly from sample archives collected from July 2004 to November 2014 with follow-up through June, 2015 at Cooperative Human Tissue Network (CHTN) Southern Division and Indivumed GmbH with prior ethical approval and patient consent for future studies. A multi-center prospective urine study to develop and validate urine biomarkers for detection of PLNM was approved by IRB at Shenzhen People’s Hospital (Shenzhen, China) (Study Number: P2014-006). Pre-biopsy fresh urine samples from the patients treated at the collaborating hospitals were collected prospectively and consecutively using a standard protocol with prior patient consent from November 2014 to June 2018 with follow-up through March, 2022. The retrospective and prospective urine studies were conducted following the Standards for Reporting Diagnostic Accuracy (STARD) guidelines and the two cohorts were described in detail previously [24]. Both studies used the same patient inclusion criteria of age at 18–90, histopathological diagnosis of PCa after urine collection, and no treatment of PCa drugs or 5-Alpha Reductase inhibitors prior to urine collection (these treatments may affect gene expression of the classifier and its ability to detect PLNM). The exclusion criteria included prostatectomy prior to urine collection. Both studies used urine samples collected without prior digital rectal examination. Patient clinicopathological information was obtained. All samples were de-identified and coded with patient numbers to protect patient privacy according to HIPAA guidelines. In both cohorts, most patients underwent pelvic lymph node dissection (PLND) following the NCCN or EAU guidelines during radical prostatectomy and the patients with PLNM were identified. The urine samples from 571 cancer patients were received in the retrospective cohort with 158 excluded (due to the lack of pathology report, diagnostic uncertainty, no PLND or PLNM data, or low/no gene expression detected), which formed IND-CHTN Cohort (*n* = 413). The urine samples from 278 patients were collected in the prospective cohort with 35 excluded due to the same reasons formed Multi-Hospital Cohort (*n* = 243). High risk PCa were defined as in the intermediate-, high- and very high-risk groups based on the NCCN risk classification guidelines (https://www.nccn.org/guidelines). In the retrospective cohort, biochemical recurrence (BCR) as defined by the NCCN guidelines was assessed every 3 months during median 8 year follow-up. In the prospective cohort, development of distant metastasis was monitored by performing imaging tests, which included computed tomography, magnetic resonance or positron emission tomography, X-ray, and bone scan, every 3 months during the median 6-year follow-up.

The urine cell sediments from 10–15 ml urine samples in the retrospective study and 15–45 ml fresh urine samples in the prospective study were collected. Urine processing, RNA purification and quantification of gene expression were performed as described previously [24] and are described in detail in Supplementary Methods.

### Development of a 25-Gene PLNM-Score classifier

To develop a gene classifier urine test with high accuracy, a random forest machine learning algorithm screening was performed by using various combinations of expression of the previously identified prostate-specific candidate genes [24] to form classifiers for distinguishing PLNM and non-PLNM. The retrospective urine cohort was used as a training set. The gene expression levels in the urine cell sediments were quantified by real-time qRT-PCR. In each gene combination, the cycle threshold (Ct) value of the genes was normalized using a housekeeping gene beta-actin (CtS = Ct(sample)/Ct(actin)). The CtS values of different genes in a combination were used by a random forest algorithm to calculate a classification score to distinguish PLNM and non-PLNM using statistical software XLSTAT (Addinsoft, Paris, France). The score was dichotomized by a cutoff value (pre-determined to be 0 by the algorithm**)** to classify the patient as PLNM (classification score ≥ 0) or non-PLNM (classification score < 0). The size of the forest was determined by the number of patients in the cohort using more than half of the patient number when each random forest algorithm was developed. A bootstrap sample randomly selected from an arbitrary subset of genes in the training data was drawn and used to develop each tree in the random forest. The classification score calculated using the random forest algorithm was compared with the PLNM diagnosis from PLND to measure the accuracy of the classification score for distinguishing PLNM and non-PLNM. By using this method, algorithms of the various gene combinations were compared to identify the algorithm with the highest accuracy. Subsequently, 10-fold cross validation was performed to calculate mean squared error of the classification algorithm with decreasing numbers of genes plotted. Genes with the lowest 10% Gini Index were excluded in each iteration. The genes with little or no contribution to the diagnostic performance of the gene combination were excluded in the final classifier. Among the algorithms of the gene combinations screened, a 25-Gene Score algorithm was found to have the highest accuracy in distinguishing PLNM and non-PLNM. The random forest parameters including mtry and nodesize were further tuned in a grid search to optimize the accuracy and form the final algorithm as the classifier. Thus, the algorithm with the cutoff value of 0 to distinguish PLNM and non-PLNM was named the 25-Gene PLNM-Score (25 G PLNM-Score) and chosen as the classifier for PLNM diagnosis. The 25 G PLNM-Score was validated in an independent prospective Multi-Hospital Cohort (*n* = 243) using the same algorithm and classification cutoff value.

### Statistical analysis

To assess the diagnostic accuracy of the 25 G PLNM-Score, the score was dichotomized using the cutoff value to classify a sample as PLNM or non-PLNM, which was then compared with the clinical diagnosis by PLND. The diagnostic performance was evaluated by univariate and multivariate discriminant analyses with measures including sensitivity, specificity, positive predictive value, negative predictive value, and their respective 95% confidence intervals. In addition, the rate of true positive, true negative, false positive and false negative was calculated. The receiver operating characteristic curve was plotted and the area under the curve (AUC) with its 95% confidence interval was calculated. The diagnostic performance of the 25 G PLNM-Score was also assessed in the high risk patients in the retrospective and prospective cohorts. Univariate and multivariate discriminant analyses were conducted to compare its diagnostic performance with ISUP/Gleason grade and cancer stage in the retrospective cohort and the Memorial Sloan Kettering Cancer Center (MSKCC) nomogram score (the MSKCC score ≥5% as PLNM and <5% as non-PLNM based on the EAU guidelines) in the prospective cohort. Kaplan–Meier plot of BCR-free survival of the patient groups stratified by the 25 G PLNM-Score, Gleason grade and cancer stage in the retrospective cohort was conducted and log rank *P* values were calculated using SPSS (IBM, Armonk, New York). Kaplan–Meier plot of distant metastasis-free survival of the patient groups stratified by the 25 G PLNM-Score and the MSKCC score in the prospective cohort was conducted with log rank P values calculated using SPSS.

## Results

### Development of a 25 G PLNM-Score Urine Test

We have previously shown that the RNA expression profiles of multiple prostate-specific biomarker candidates involved in cancer tumorigenesis, progression and metastasis in the urine cell pellets correlated to the gene expression patterns in the prostate tumor specimens and could be used as gene panel-based urine tests for PCa diagnosis and prognosis [24–26]. A random forest machine learning algorithm is typically used to assemble the selected features/variables into a classifier [27, 28]. To develop a urine gene panel-based machine learning algorithm as a classifier for distinguishing PLNM and non-PLNM with high accuracy, we conducted a random forest machine learning algorithm screening using various combinations of the prostate-specific candidate genes in a multi-center retrospective urine cohort (IND-CHTN Cohort) as training set (Supplementary Fig. S1). The gene expression level was quantified by real-time qRT-PCR in the urine cell pellets collected without prior digital rectal examination (DRE) [24]. 20 out of 413 patients in the cohort had PLNM as diagnosed by PLND. The median number of lymph nodes dissected was 6 (Q1, Q3: 4, 10) (Table 1). The accuracy of the gene-panel scores calculated by machine learning algorithms of various combinations of the candidate genes was assessed and compared. A 25-Gene Score, which was based on RNA expression of *HIF1A, FGFR1, BIRC5, AMACR, CRISP3, FN1, HPN, MYO6, PSCA, PMP22, GOLM1, LMTK2, EZH2, GSTP1, PCA3, VEGFA, CST3, PTEN, PIP5K1A, CDK1, TMPRSS2, ANXA3, CCNA1, CCND1*, and *KLK3*, was found to exhibit the highest accuracy and was chosen as the classifier for diagnosis of PLNM in urine samples (named 25 G PLNM-Score).

**Table 1:** Patient characteristics.

	IND-CHTN Retrospective Cohort	Multi-Hospital Prospective Cohort
No of patients	413	243
Median age (Q1, Q3)	65 (61, 68)	70 (64, 76)
ISUP/Gleason grade group (%)		
Group 1: ≤6 ( ≤ 3 + 3)	76 (18%)	56 (23%)
Group 2: 7 (3 + 4)	197 (48%)	47 (19%)
Group 3: 7 (4 + 3)	113 (27%)	63 (26%)
Group 4: 8 (4 + 4, 3 + 5, 5 + 3)	9 (2%)	43 (18%)
Group 5: 9 or 10 (4 + 5, 5 + 4, or 5 + 5)	18 (4%)	34 (14%)
Pre-operative PSA (%)		
PSA < 10 ng/dL	0	84 (35%)
PSA 10-20 ng/dL	0	53 (22%)
PSA > 20 ng/dL	0	104 (43%)
PSA unknown	413 (100%)	2 (0.8%)
Pelvic lymph node metastasis (%)	20 (5%)	35 (14%)
Median number of lymph nodes dissected (Q1, Q3)	6 (4, 10)	13 (9, 15)
Distant metastasis (%)	9 (2%)	76 (31%)
BCR (%)	42 (10%)	-
Cancer risk (%)		
Low risk	42 (10%)	29 (12%)
High risk	371 (90%)	214 (88%)

*PSA* prostate specific antigen, *BCR* biochemical recurrence.

**Fig. 1:**
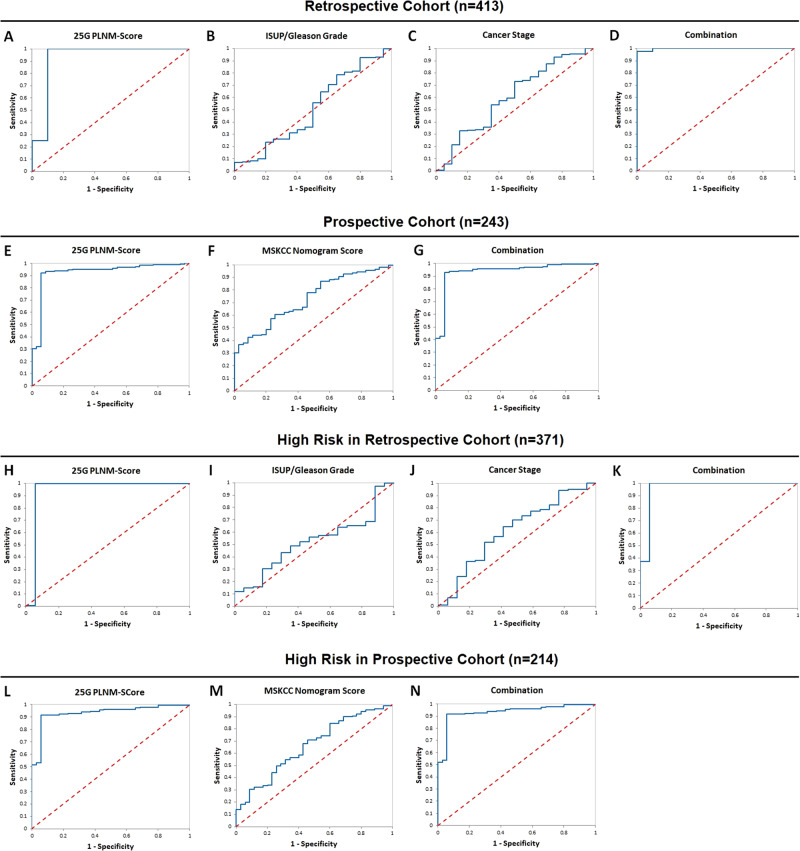
Analyses of the diagnostic performance of the 25 G PLNM-Score as a biomarker. Receiver operating characteristic (ROC) curves in the retrospective cohort and prospective cohort are shown. The 25-Gene PLNM-Score urine test (25 G PLNM-Score) and the clinical parameters are assessed for their performance to identify pelvic lymph node metastasis in these cohorts. ROC curves of the 25 G PLNM-Score (**A**), ISUP/Gleason grade (**B**), cancer stage (**C**), and their combination (**D**) in the retrospective IND-CHTN Cohort. ROC curves of the 25 G PLNM-Score (**E**), the MSKCC nomogram score (**F**), and their combination (**G**) in the prospective Multi-Hospital Cohort. ROC curves of the 25 G PLNM-Score (**H**), ISUP/Gleason grade (**I**), cancer stage (**J**), and their combination (**K**) in the high risk patients in the retrospective IND-CHTN Cohort. ROC curves of the 25 G PLNM-Score (**L**), the MSKCC nomogram score (**M**), and their combination (**N**) in the high risk patients in the prospective Multi-Hospital Cohort.

The diagnostic performance of the 25 G PLNM-Score was measured by univariate discriminant analysis and the result showed high accuracy with a sensitivity of 90% (95% CI: 77–103%), specificity of 100% (95% CI: 100–100%), and AUC of 0.93 (95% CI: 0.85–1.01) (Table 2, Fig. 1A). For comparison, the ability of ISUP/Gleason grade and cancer stage to distinguish PLNM and non-PLNM in the cohort was tested and the result showed extremely low specificity and AUC (Table 2, Fig. 1B, C). Interestingly, when they were combined with the 25 G PLNM-Score in multivariate discriminant analysis, the accuracy increased with sensitivity and AUC reaching 100% (95% CI: 100–100%) and 1.00 (95% CI: 0.98–1.01) respectively (Table 2, Fig. 1D). This suggests that the 25 G PLNM-Score may be combined with ISUP/Gleason grade and cancer stage to provide highly accurate detection of PLNM.

**Table 2:** Diagnostic performance of the 25-Gene PLNM-Score urine test (25 G PLNM-Score) for detecting pelvic lymph node metastasis (PLNM) in a retrospective IND-CHTN Cohort and a prospective Multi-Hospital Cohort.

	Sensitivity (95% CI)	Specificity (95% CI)	PPV (95% CI)	NPV (95% CI)	AUC (95% CI)
PLNM detection in the IND-CHTN Cohort (*n* = 413)					
25 G PLNM-Score	90% (77–103%)	100% (100–100%)	100% (100–100%)	99% (99–100%)	0.93 (0.85–1.01)
ISUP/Gleason grade	100% (100–100%)	6.6% (4.2–9.1%)	5.2% (3.0–7.4%)	100% (100–100%)	0.51 (0.38–0.64)
Cancer stage	100% (100–100%)	0.25% (−0.24–0.75%)	4.9% (2.8–6.9%)	100% (100–100%)	0.60 (0.47–0.74)
Combination	100% (100–100%)	97% (96–99%)	65% (48–81%)	100% (100–100%)	1.00 (0.98–1.01)
PLNM detection in the Multi-Hospital Cohort (*n* = 243)					
25 G PLNM-Score	94% (87–102%)	92% (89–96%)	67% (54–80%)	99% (98–100%)	0.93 (0.87–0.99)
The MSKCC Score	100% (100–100%)	17% (12–22%)	17% (12–22%)	100% (100–100%)	0.73 (0.63–0.83)
Combination	100% (100–100%)	17% (12–22%)	17% (12–22%)	100% (100–100%)	0.94 (0.88–0.99)

*CI* confidence interval, *PPV* positive predictive value, *NPV* negative predictive value, *AUC* area under the curve, *PSA* prostate specific antigen.

### Validation of the 25 G PLNM-score urine test

The 25 G PLNM-Score was validated in an independent multi-center prospective Multi-Hospital Cohort (*n* = 243), in which 35 patients were found to have PLNM by PLND. The median number of lymph nodes dissected was 13, which was higher than that in the retrospective cohort (Table 1). As in the retrospective cohort, urine samples were collected without DRE, and the same diagnostic algorithm and cutoff value were used with the quantities of the 25 genes in the urine cell pellets to calculate the 25 G PLNM-Score. The established MSKCC nomogram score for stratifying PLNM and non-PLNM in clinical practice was also assessed in the cohort as a comparison. The result showed similarly high accuracy of the 25 G PLNM-Score with a sensitivity of 94% (95% CI: 87–102%), specificity of 92% (95% CI: 89–96%), and AUC of 0.93 (95% CI: 0.87–0.99), while the MSKCC score had extremely low specificity and AUC [17% (95% CI: 12–22%) and 0.73 (95% CI: 0.63–0.83) respectively] (Table 2, Fig. 1E, F). When they were combined, the accuracy did not increase (Table 2, Fig. 1G). These results showed that the 25 G PLNM-Score could accurately detect PLNM to overcome the limited accuracy of the MSKCC score.

### Performance in high risk patients

In clinical practice, high risk patients (including intermediate-, high- and very high-risk according to the NCCN guidelines) typically receive PLND during RP, which underscores the importance of accurately selecting patients for PLND in these patients. Thus, the accuracy of the 25 G PLNM-Score to detect PLNM in high risk patients was tested. It showed similarly high diagnostic accuracy with a sensitivity of 94% (95% CI: 83–105%), specificity of 100% (95% CI: 100–100%), and AUC of 0.94 (95% CI: 0.86–1.02) in the retrospective cohort, and a sensitivity of 94% (95% CI: 87–102%), specificity of 92% (95% CI: 88–96%), and AUC of 0.93 (95% CI: 0.87–0.99) in the prospective cohort (Supplementary Table S1, Fig. 1H, L). In contrast, ISUP/Gleason grade and cancer stage had extremely low specificity and AUC in the retrospective cohort, and the MSKCC score had extremely low specificity and AUC in the prospective cohort (Supplementary Table S1, Fig. 1I, J, M). Combining these factors with the 25 G PLNM-Score did not improve the diagnostic accuracy (Supplementary Table S1, Fig. 1K, N).

### Prognosis for BCR and distant metastasis

**Fig. 2:**
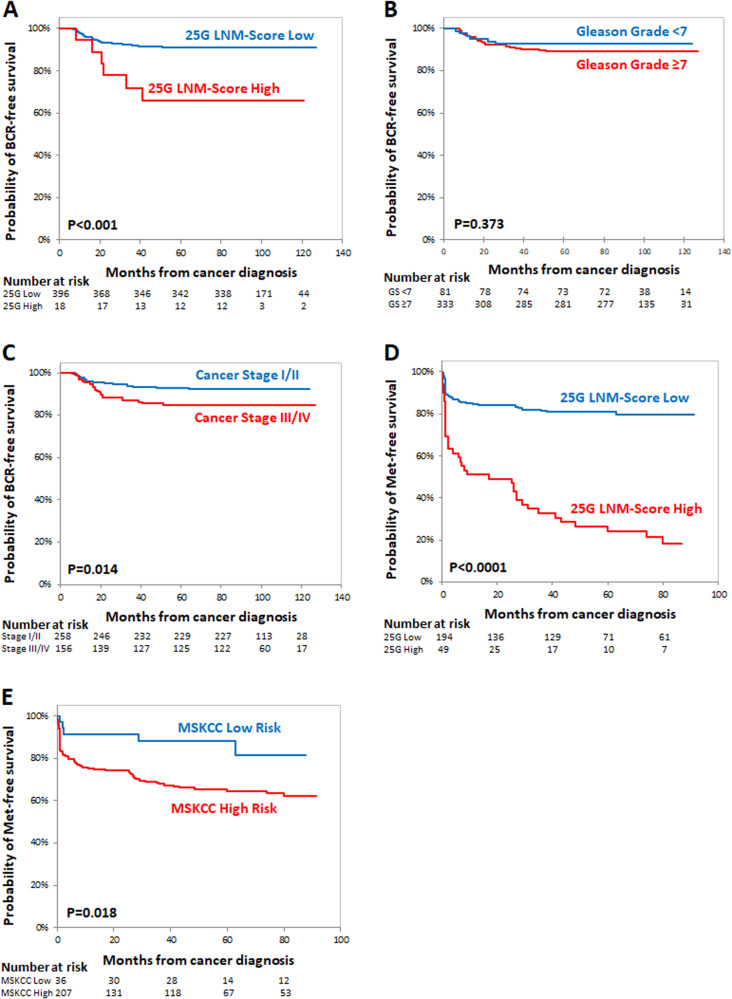
Analyses of the 25-Gene PLNM-Score as a biomarker to predict the distant metastasis in the patient cohorts. Kaplan–Meier survival plot of biochemical recurrence (BCR)-free survival and distant metastasis-free survival of the 25-Gene PLNM-Score low and high groups in the retrospective IND-CHTN Cohort and prospective Multi-Hospital Cohort. Kaplan–Meier survival curves of the 25-Gene PLNM-Score (**A**), ISUP/Gleason grade (**B**), and cancer stage (**C**) for predicting BCR-free survival in the IND-CHTN Cohort. Kaplan–Meier survival curves of the 25-Gene PLNM-Score (**D**) and the MSKCC nomogram score (**E**) for predicting distant metastasis-free survival in the Multi-Hospital Cohort. Log rank *P* values are shown.

In the retrospective cohort, 42 patients had recurrence after treatment during the median 8 year follow-up (Table 1). The median BCR-free survival time was 89 (Q1, Q3: 31, 98) months for the patients with high 25 G PLNM scores (above the cutoff value) and 98 (90, 109) months for the patients with low 25 G PLNM scores (below the cutoff value). Kaplan–Meier plot of BCR-free survival showed large and statistically significant difference in the patients stratified by the 25 G PLNM-Score (log rank *P* < 0.001) (Fig. 2A). In contrast, the median BCR-free survival time was similar between the ISUP/Gleason grade <7 and ≥7 groups [99 (91, 113) months and 97 (89, 107) months, respectively] (log rank *P* = 0.373) and cancer stage I/II and III/IV groups [98 (90, 109) months and 96 (88, 108) months respectively] (log rank *P* = 0.014) (Fig. 2B, C).

Since the development of distant metastasis has more significant impact on PCa progression, treatment, and mortality, we tested if the patients stratified by the 25 G PLNM-Score had different outcome in the development of distant metastasis. In the prospective cohort, 76 patients (31%) developed distant metastasis during the median 6-year follow-up (Table 1). The median distant metastasis-free survival time was different among the patients with high [17 (Q1, Q3: 1, 53) months] and low [54 (13, 82) months] 25 G PLNM scores. Kaplan–Meier plot of metastasis-free survival showed large and statistically significant difference in the patients stratified by the 25 G PLNM-Score (log rank *P* < 0.0001) (Fig. 2D). In contrast, the median metastasis-free survival time was similar among the low [54 (40, 84) months] and high [50 (7, 81) months] MSKCC score groups (<5% vs ≥5%). A smaller yet statistically significant difference in metastasis-free survival was found in the patients stratified by the MSKCC score (log rank *P* = 0.018) (Fig. 2E).

### Potential clinical benefits

PLNM is an important determining factor in treatment decision-making, therefore, it is crucial to accurately identify PLNM, especially in high risk patients. The rates of true and false positive (TP, FP), and true and false negative (TN, FN) of the 25 G PLNM-Score in the diagnosis of PLNM were calculated and compared with ISUP/Gleason grade and cancer stage in the retrospective cohort and the MSKCC score in the prospective cohort respectively (Table 3). In the retrospective cohort, the 25 G PLNM-Score had much higher TP rate while achieving much lower FP rate as compared to ISUP/Gleason grade and cancer stage (100%, 5.2%, 4.9% TP rate, respectively, 0%, 95%, 95% FP rate respectively). More importantly, using the 25 G PLNM-Score to detect PLNM would spare 96% of patients (395/413) from unnecessary PLND with only 0.51% of PLNM patients (2/395) missing PLND, as compared to 6.3% (26/413) of patients spared with 0% (0/26) missing by ISUP/Gleason grade, and 0.24% (1/413) spared with 0% (0/1) missing by cancer stage. In the prospective cohort, the 25 G PLNM-Score had much higher TP rate than the MSKCC score (67% and 17%, respectively). More significantly, the 25 G PLNM-Score spared 80% of patients (194/243) from PLND with only 1% of patients (2/194) missing, while the MSKCC score could only spare 15% (36/243) with 0% (0/36) missing (Table 3). The consistent results in both cohorts showed potentially large clinical benefit by using the 25 G PLNM-Score to select patients with PLNM for PLND/eLND.

**Table 3:** Clinical benefit of using the 25-Gene PLNM-Score urine test (25 G PLNM-Score) for detecting pelvic lymph node metastasis (PLNM) in the retrospective IND-CHTN Cohort and prospective Multi-Hospital Cohort.

	Positive Diagnosis	Negative Diagnosis	Total	TP Rate (%)	TN Rate (%)	FP Rate (%)	FN Rate (%)	PLND Spared (%)	PLNM Missed (%)
25 G PLNM-Score diagnosis in the IND-CHTN Cohort (*n* = 413)									
PLNM	18	2	20	18/18 (100%)	393/395 (99%)	0/18 (0%)	2/395 (0.51%)	395/413 (96%)	2/395 (0.51%)
Non-PLNM	0	393	393						
ISUP/Gleason grade diagnosis in the IND-CHTN Cohort (*n* = 413)									
PLNM	20	0	20	20/387 (5.2%)	26/26 (100%)	367/387 (95%)	0/26 (0%)	26/413 (6.3%)	0/26 (0%)
Non-PLNM	367	26	393						
Cancer stage diagnosis in the IND-CHTN Cohort (*n* = 413)									
PLNM	20	0	20	20/412 (4.9%)	1/1 (100%)	392/412 (95%)	0/1 (0%)	1/413 (0.24%)	0/1 (0%)
Non-PLNM	392	1	393						
25 G PLNM-Score diagnosis in the Multi-Hospital Cohort (*n* = 243)									
PLNM	33	2	35	33/49 (67%)	192/194 (99%)	16/49 (33%)	2/194 (1%)	194/243 (80%)	2/194 (1%)
Non-PLNM	16	192	208						
The MSKCC score diagnosis in the Multi-Hospital Cohort (*n* = 243)									
PLNM	35	0	35	35/207 (17%)	36/36 (100%)	172/207 (83%)	0/36 (0%)	36/243 (15%)	0/36 (0%)
Non-PLNM	172	36	208						

*TP* true positive, *TN* true negative, *FP* false positive, *FN* false negative, *PLND* pelvic lymph node dissection, *PLNM* pelvic lymph node metastasis.

## Discussion

In this study, we developed and validated a novel, non-invasive, machine learning algorithm-based 25 G PLNM-Score for detecting PLNM in newly diagnosed PCa patients with high accuracy in two independent, multi-center retrospective and prospective urine cohorts using urine samples collected without DRE. In addition, the 25 G PLNM-Score could accurately identify PLNM in the high risk patients. In contrast, the MSKCC score and clinicopathological factors such as ISUP/Gleason grade and cancer stage could not accurately detect PLNM. Furthermore, the patients stratified by the 25 G PLNM-Score had marked difference in cancer recurrence and the development of distant metastasis. The study clearly demonstrated a significant clinical benefit of using the 25 G PLNM-Score to accurately select PLNM patients for PLND while sparing the non-PLNM patients from unnecessary surgery and potential side effects.

The accuracy of the 25 G PLNM-Score in the retrospective and prospective cohorts were similarly high, even if the two cohorts used urine samples collected differently as frozen urine pellets after long-term storage (retrospective cohort) or freshly collected urine (prospective cohort). The result showed that the test was robust and could be used in different clinical situations. In addition, the 25-Gene panel with a different algorithm/cutoff value was found to be able to accurately identify PLNM using prostate tissue specimens in several biopsy/RP cohorts (unpublished data). This suggests a strong correlation of RNA expression of the 25 genes detected in the urine test with that in prostate biopsy specimens.

Currently, none of the clinicopathological parameters (such as ISUP/Gleason grade, cancer stage, pre-operative PSA), nomograms (such as the MSKCC score, Roach formula, Briganti score, Partin tables), and various imaging tools (MRI, CT scan, PSMA PET/CT, mpMRI) could detect PLNM with high precision. None of the test had sensitivity and specificity above 90%, and AUC over 0.9 [12, 14–19, 21–23]. The recent development combining machine learning assessment with imaging measurements to improve the predictive power of PLNM showed promise, yet most tests are costly and cannot reach high accuracy. Although one model combining mpMRI assessed by machine learning with clinicopathological factors showed high AUC in the development and internal validation test, the external validation showed very low AUC [20]. In contrast, our 25 G PLNM-Score showed consistently high diagnostic sensitivity and specificity above 90% and AUC exceeding 0.9 in two independent multi-center studies. Its direct side-by-side comparison with the MSKCC score corroborated with its superior diagnostic power. This suggests that the 25 G PLNM-Score may be a more accurate and better diagnostic tool than all existing methods. In addition, our study found that it could be combined with ISUP/Gleason grade and cancer stage to provide exceptionally accurate diagnosis in the retrospective cohort. Thus, it may be combined with existing PLNM tools to greatly improve diagnosis accuracy and avoid unnecessary PLND.

In this study, we showed that the 25 G PLNM-Score was able to stratify patients with significant difference in BCR-free survival. More importantly, we found that the 25 G PLNM-Score could accurately predict the incidence of distant metastasis during long-term follow-up and the patients with high 25 G PLNM scores developed more distant metastasis with much shorter metastasis-free survival time than the patients with low 25 G PLNM scores. In contrast, high and low MSKCC score had similar metastasis-free survival time with little ability to predict metastasis. The result that stratification of the patients by the 25 G PLNM-Score could accurately separate the patients with or without distant metastatic risk further demonstrated the validity of using it to stratify PLNM and non-PLNM patients. Such stratification can provide better and more meaningful clinical guidance for PLND and subsequent treatment decision-making than the existing PLNM tests. Our study is the first test linking PLNM stratification to prediction of distant metastasis, and the 25 G PLNM-Score was the first test capable of identifying PLNM with metastatic potential for treatment decision-making.

It is of great clinical benefit to accurately identify PLNM patients before PLND/eLND to avoid unnecessary surgery for non-PLNM patients. Although several nomograms and imaging-based detection methods have been used in clinical practice, their diagnostic accuracy and clinical benefit are limited. In our study, the MSKCC score could only spare 17% of patients undergoing PLND. Other nomograms including Roach formula, Briganti score and Partin tables had been shown to have similar accuracy as the MSKCC score [14–16]. A PLNM-Risk model combining mpMRI assessed by machine learning with clinicopathological factors was shown to have 59.6% of ePLNDs spared with 1.7% of PLNM missing [20]. The 2019 Briganti nomogram at 7% cutoff spared 56% of ePLNDs with 2.6% of PLNM missing [23]. In contrast, our 25 G PLNM-Score spared 96% of PLND with 0.51% of PLNM missing in the retrospective cohort, and spared 80% of PLND with 1% of PLNM missing in the prospective cohort. This demonstrated that the 25 G PLNM-Score has a more significant clinical benefit than the existing tests by reducing higher number of unnecessary PLND and potentially serious side effects with smaller risk of missing PLNM patients.

Previously, we identified a 25-Gene Panel for PCa diagnosis [24]. Although the 25 G PLNM-Score uses the same 25 genes, its algorithm for diagnosis of PLNM is completely different from that used by the 25-Gene Panel for PCa diagnosis. The RNA expression levels of the 25 genes coupled with different algorithms can potentially be used for both cancer screening/diagnosis and PLNM detection to improve cancer diagnosis and treatment.

The samples in the prospective validation cohort were collected from the Chinese patients and the samples in the retrospective development cohort were obtained from US and Europe with mostly Caucasian patients. The similar accuracy of the 25 G PLNM-Score in detecting PLNM in the two cohorts suggests that the test is robust and may be used in different patient populations regardless of race.

The limitations of this study included that no MSKCC score was available for comparison in the retrospective cohort, and no imaging data was available for a direct comparison with the 25 G PLNM-Score in both cohorts. The number of patients who had pre-surgical MRI was 39 out of 413 (9%) in the retrospective cohort and 52 out of 243 (21%) in the prospective cohort. Although we did not have the imaging data to assess the ability of MRI to detect PLNM in our cohorts, it’s not critical as numerous publications have already shown that various imaging tests including MRI had limited accuracy in PLNM detection. For example, mpMRI had low sensitivity of 40–60% [17, 21], combining imaging technologies with clinicopathological factors resulted in improved yet still limited accuracy such as AUC of 0.79 [20, 22, 23]. Our results showed high accuracy of the 25 G PLNM-Score in the two cohorts, and comparison with the MSKCC score and clinicopathological factors showed its superior performance. The lack of comparison with an imaging test did not impact our findings. In addition, the retrospective and prospective cohorts have differences in clinicopathological characteristics, such as the % of ISUP/Gleason grade group 4–5 patients (6% in the retrospective cohort vs 42% in the prospective cohort), the % of PLNM (5% vs 14%), and median age at diagnosis (65 vs 70) (Table 1), which may affect proper validation of the 25 G PLNM-Score. Thus, large studies with more PLNM patients in different cohorts will be conducted in the future to further validate the 25 G PLNM-Score and compare its diagnostic performance with different nomograms and imaging-based tests.

In summary, we developed and validated a highly accurate and non-invasive machine learning algorithm-based 25 G PLNM-Score urine test, which can be used to identify PLNM patients for PLND/eLND and non-PLNM patients for avoiding unnecessary surgery and serious side effects. Its clinical application may potentially benefit treatment decision-making in newly diagnosed prostate cancer patients.

## Supplementary information

Revised Supplementary Data

## Data Availability

The data underlying this article will be shared on reasonable request to the corresponding author.
