# Supplementary material for: A non-invasive 25-Gene PLNM-Score urine test for detection of prostate cancer pelvic lymph node metastasis
Source: Prostate Cancer Prostatic Dis. 2024 Feb 2;28(1):94–102. doi: 10.1038/s41391-023-00758-z (PMC11860222; doi:10.1038/s41391-023-00758-z)

**A non-invasive 25-Gene LNM-Score urine test for detection of prostate cancer pelvic lymph node metastasis**

Supplementary Data

**Methods**

**Urine Sample Processing and Quantification of Gene Expression**

The urine processing and gene expression quantification were performed as described (24). Briefly, 10–15 ml urine samples obtained without digital rectal examination (DRE) in the retrospective study were pelleted by centrifugation and the flash-frozen pellets were stored at -80°C. In the prospective study, 15–45 ml urine sample without DRE was collected in a collection tube containing 5 ml DNA/RNA preservative AssayAssure (Thermo Fisher Scientific, Waltham, MA, USA) or U-Preserve (Hao Rui Jia Biotech Ltd., Beijing, China) and stored at 4°C for 1-7 days. The urine sample was centrifuged at 1000×g for 10 min followed by washing with phosphate-buffered saline (PBS) and a second centrifugation to collect cell pellet. The pellet was processed for RNA purification or frozen on dry ice and stored at -80°C until RNA purification.

In the frozen urine pellet, RNA was purified using Quick-RNA MicroPrep Kit (Zymo Research, Irvine, CA, USA) followed by cDNA reverse transcription using either High Capacity cDNA Reverse Transcription Kit (Life Technologies, Foster City, CA, USA) or iScript Reverse Transcription Supermix for Real Time qRT-PCR (Bio-Rad, Hercules, CA, USA). cDNA preamplification was performed using either TaqMan® PreAmp Master Mix (Thermo Fisher Scientific, Waltham, MA, USA) or PCa PreAmplification Mix (Hao Rui Jia Biotech Ltd., Beijing, China). mRNA quantification was performed by real-time qRT-PCR with predesigned primers and probe assays from Integrated DNA Technologies (San Diego, CA, USA) using TaqMan® Universal PCR Master Mix (Thermo Fisher Scientific, Waltham, MA, USA) or PrimeTime® Gene Expression Master Mix (Integrated DNA Technologies, San Diego, CA, USA). For each gene, triplicate PCRs were performed on ABI Quantstudio 6, ABI 7500 or ABI 7900 Real-Time PCR System (Thermo Fisher Scientific, Waltham, MA, USA). The gene expression data was initially analyzed using ABI Quantstudio 6, ABI 7500 or ABI 7900 software. The mRNA level of a housekeeping gene beta-actin was measured in each urine sample and used to normalize expression of each gene in the classifier. The cycle threshold (Ct) value of each gene was divided by the Ct value of the beta-actin as the normalized gene expression value (CtS=Ct(sample)/Ct(actin)). Mean Ct value from triplicate PCRs was used for calculation.

**Supplementary Tables**

**Supplementary Table S1.** Diagnostic performance of the 25-Gene PLNM-Score urine test (25G PLNM-Score) for detecting pelvic lymph node metastasis in high risk patients in a retrospective IND-CHTN Cohort and a prospective Multi-Hospital Cohort.

|  | Sensitivity  (95% CI) | Specificity  (95% CI) | PPV  (95% CI) | NPV  (95% CI) | AUC  (95% CI) |
| --- | --- | --- | --- | --- | --- |
| **PLNM detection in the IND-CHTN Cohort (n =371)** | | | | | |
| 25G PLNM-Score | 94%  (83-105%) | 100%  (100-100%) | 100%  (100-100%) | 100%  (99-100%) | 0.94  (0.86-1.02) |
| ISUP/Gleason grade | 100%  (100-100%) | 5.7%  (3.2-8.1%) | 4.8%  (2.6-7.1%) | 100%  (100-100%) | 0.52  (0.38-0.66) |
| Cancer stage | 100%  (100-100%) | 0.28%  (-0.27-0.84%) | 4.6%  (2.5-6.7%) | 100%  (100-100%) | 0.62  (0.47-0.76) |
| Combination | 100%  (100-100%) | 36%  (31-41%) | 7.0%  (3.8-10%) | 100%  (100-100%) | 0.96  (0.90-1.03) |
| **PLNM detection in the Multi-Hospital Cohort (n = 214)** | | | | | |
| 25G PLNM-Score | 94%  (87-102%) | 92%  (88-96%) | 69%  (56-82%) | 99%  (97-100%) | 0.93  (0.87-0.99) |
| The MSKCC Score | 100%  (100-100%) | 0.50%  (1.8-8.2%) | 17%  (12-22%) | 100%  (100-100%) | 0.66  (0.56-0.77) |
| Combination | 94%  (87-102%) | 92%  (88-96%) | 69%  (56-82%) | 99%  (97-100%) | 0.93  (0.87-0.99) |
| CI = confidence interval; PPV = positive predictive value; NPV = negative predictive value; AUC = area under the curve. | | | | | |

**Supplementary Figures**

**Supplementary Figure S1.** Study design.


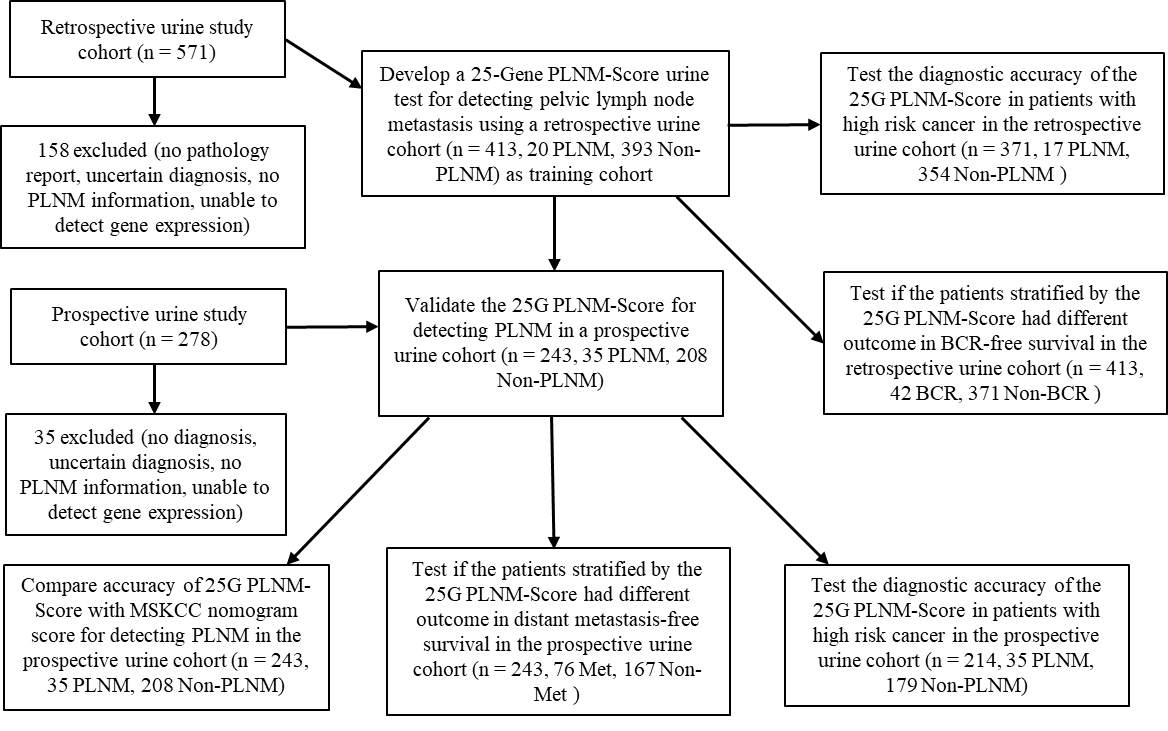

Supplement: Supplementary file 1 — Revised Supplementary Data [file 41391_2023_758_MOESM1_ESM.docx]
